# Peak Net Pressure Coefficients of Elliptical Center-Open Dome Roofs

**DOI:** 10.3390/ma15165497

**Published:** 2022-08-10

**Authors:** Jong Ho Lee, Dong Jin Cheon, Yong Chul Kim, Sung Won Yoon

**Affiliations:** 1 Department of Architecture, Seoul National University of Science and Technology, 232, Gongneung-ro, Nowon-gu, Seoul 01811, Korea; 2 Department of Architecture, Tokyo Polytechnic University, Atsugi 243-0297, Kanagawa, Japan

**Keywords:** elliptical retractable dome roof, wind pressure coefficient, cladding design, wind tunnel test, wind load code, peak net pressure

## Abstract

Recently, the demand for spatial structures such as retractable dome roofs is increasing. The safety of dome roofs must be ensured even when they are open. Hence, studies analyzing the peak pressure coefficients of spherical dome roofs are actively being conducted. However, no peak pressure coefficients for the cladding design of elliptical retractable dome roofs have been proposed. Although several studies on elliptical open dome roofs that open from the edge to the center have been conducted, studies on those that open from the center to the edge are still insufficient. This study investigated the peak pressure coefficients of elliptical center-open dome roofs. For wind tunnel tests, a model was fabricated with an opening ratio of 30%. Under experimental conditions, five different wall height-to-span ratios (from 0.1 to 0.5) were used, with the roof rise-to-span ratio set at 0.1. Accordingly, the experimental values of the peak pressure coefficients of elliptical center-open dome roofs were compared with those of the closed dome roofs proposed in the Korean and Japanese wind load codes. Subsequently, their efficiency was verified. The findings were also compared with previous research outcomes. Based on the results, peak net pressure coefficients are proposed for cladding designs suitable for elliptical center-open dome roofs.

## 1. Introduction

Following the recent rising demand for indoor leisure activities and sports, the number of retractable dome roofs is increasing worldwide. Compared with closed dome roofs, retractable dome roof structures can operate in open, partially open, and closed states, thus allowing them to flexibly adapt to seasonal and weather conditions. Moreover, owing to their structural efficiency and economic advantages, they are mostly used in large spatial structures such as stadiums. As dome roofs primarily consist of lightweight materials, such as membranes, they are particularly sensitive to wind pressure, which can tear or damage the roof. Dome roofs in numerous countries, including the ones in the Jeju Soccer Field, the Incheon Munhak, Sultan Mizan Zainal Abidin, and Montreal Olympic Stadium, have suffered damages and malfunctions as a result of wind pressure. These accidents were primarily a result of the failure to predict the wind pressure on the cladding due to strong winds, leading to local or global damages [1]. 

As retractable dome roofs can be opened and closed, loads must be considered in both the opened and closed states. In the basic design stage, the geometry or structural elements are often determined based on the wind load code. The design modifications and retesting at this stage can consume time and economic resources. However, at present, no peak pressure coefficients for cladding designs have been proposed for retractable dome roofs. Moreover, related studies are few [2,3,4].

The complex geometric problems of retractable dome roofs have not been completely resolved. Recently, studies on retractable dome roofs with circular shapes have been conducted. However, elliptical dome roofs account for a greater proportion of retractable dome roofs that have been built or are under construction. As elliptical dome roofs have a longitudinal and transverse axis, a more detailed analysis is necessary.

Concerning studies on retractable spherical dome roofs, Cheon et al. [5] analyzed the peak pressure coefficients through wind tunnel tests and compared them with AIJ–RLB (2015) [6], the Japanese wind load code. According to their results, the experimental value exceeded the code in an open state. 

Kim et al. [7] analyzed the peak pressure coefficients of a retractable spherical dome roof through wind tunnel tests, compared them with AIJ–RLB (2015), the Japanese wind load code, and proposed peak pressure coefficients for cladding design. 

Cheon et al. [8] and Park et al. [9] analyzed peak pressure coefficients and peak net pressure coefficients for a center-open dome roof through wind tunnel tests, compared them with the Japanese wind load code (AIJ–RLB (2015)), and proposed peak net pressure coefficients. 

Active studies on elliptical retractable dome roofs have been conducted recently. Lee et al. [10] analyzed the peak pressure coefficients of a dome roof that opens from the edge to the center based on wind tunnel tests and compared the findings with the Japanese wind load code (AIJ–RLB (2015)). The outcomes demonstrated that the experimental values exceeded those obtained using the load code. Further, Lee et al. [11] proposed peak pressure coefficients for the cladding design of elliptical retractable dome roofs. 

However, studies on the wind pressure coefficients of elliptical center-open dome roofs and on the peak net pressure coefficient, which denotes the difference between the external and internal roof surface pressures and the inner surface wind pressure caused by the inflow of air from the open roof, respectively, are few.

For open roofs, wind pressure fluctuations caused by changes in airflow inside the structures should be considered. However, as current codes do not consider open roofs, the peak pressure coefficients of the internal roof surfaces cannot be analyzed.

This study analyzed the peak pressure coefficients and peak net pressure coefficients of an elliptical center-open dome roof based on wind tunnel tests, compared the experimental results with previous research based on the Japanese (AIJ–RLB (2015)) and the Korean wind load codes (KDS 41 10 15) [12], examined the adequacy of the codes, and proposed suitable peak net pressure coefficients for the cladding design of elliptical, retractable dome roofs.

## 2. Wind Tunnel Tests

### 2.1. Model Details

**Figure 1:**
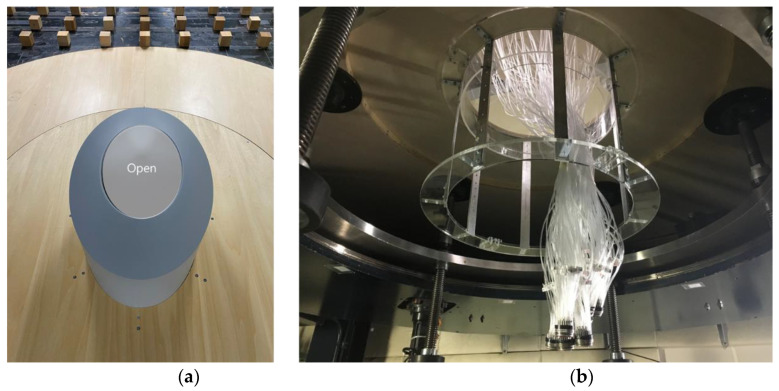
(**a**) Test dome model (opening ratio of 30%) and (**b**) installed pressure taps.

Center-open dome roofs open in the direction of the center to the edge. The model was fabricated with an opening ratio of 30%, where the opening ratio was defined according to the open area of the roof. Figure 1a shows the model, with the central shaded area open and the remaining edges surrounded by the roof.

A total of 80 pressure taps were installed in four lines at 30° intervals, with 10 external and 10 internal taps in each line. Table 1 lists the detailed number of pressure taps on the outer and inner sides of the roof. Each pressure tap installed on the model has a length of 900 mm and an internal diameter of 1.4 mm, as shown in Figure 1b.

### 2.2. Wind Tunnel Test Conditions and Methods

**Figure 2:**
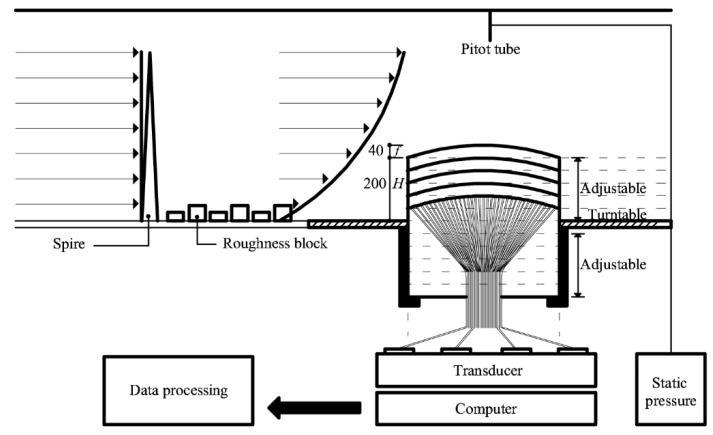
Schematic of the wind tunnel test.

Figure 2 shows the outline of the wind tunnel test, which was conducted in a large boundary-layer wind tunnel located at Tokyo Polytechnic University, Japan. Its working section was 1.8 m × 2.2 m (height × width). By considering a full-scale dome with a 72-m longitudinal axis, 48-m transverse axis, and height in the range of 0–30 m and applying a length scale of 1/150, the test dome model consisted of a 0.48-m longitudinal axis, 0.32-m transverse axis, and height in the range of 0–0.2 m. The length ratio of the longitudinal and transverse axes was 1:1.5 [1].

As the blockage ratio was less than 2%, data correction was not required. The roof rise-to-span ratio (*f*/*D*) was 0.1, and the roof rise was 0.04 m. According to Ishii [13], the *f*/*D* values of constructed open-dome roofs range from 0 to 0.2. The test was performed at five different heights, the wall height-to-span ratio (*h*/*D*) was varied from 0.1 to 0.5 at 0.1 intervals, and the wall height (*h*) was increased at 0.04 m increments.

**Figure 3:**
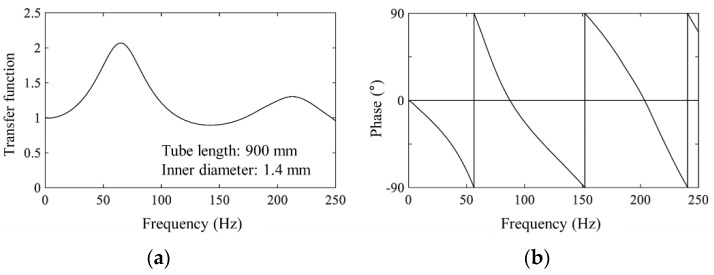
Function for tubing effects corrections: (**a**) transfer function and (**b**) phase difference.

All the pressures were measured simultaneously using a multichannel pressure measurement system. The sampling frequency was 1000 Hz. The tubing effects were numerically corrected using a transfer function, and the phase difference of the pressure measurement system with a cut-off frequency of 250 Hz was determined using low-pass filtering, as shown in Figure 3 [14].

**Figure 4:**
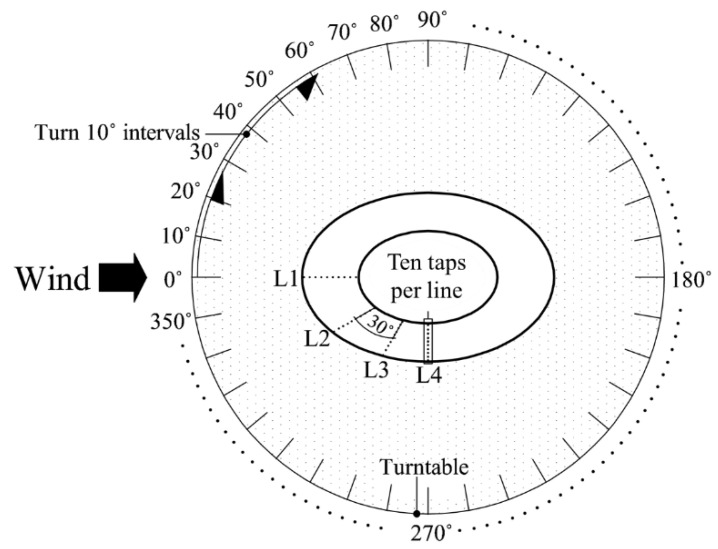
The 36 wind directions considered for test and locations of pressure taps.

As shown in Figure 4, the test was conducted at a total of 36 wind directions from 0° to 350° at intervals of 10°. Although only four pressure taps were installed at 0°, 30°, 60°, and 90° each, the data obtained from the pressure taps of the four lines were analyzed using the corresponding symmetric value of the overall wind direction. 

### 2.3. Experimental Flows

The test was conducted under the same conditions to compare the results with the Japanese wind load code (AIJ–RLB (2015)) for dome roofs and the external peak pressure coefficients for cladding designs proposed in previous studies. The wind speed scale was 1/3 and the time scale was 1/50; thus, the actual time of 10 min was 12 s in the wind tunnel, and the moving average time was 1 s. 

**Figure 5:**
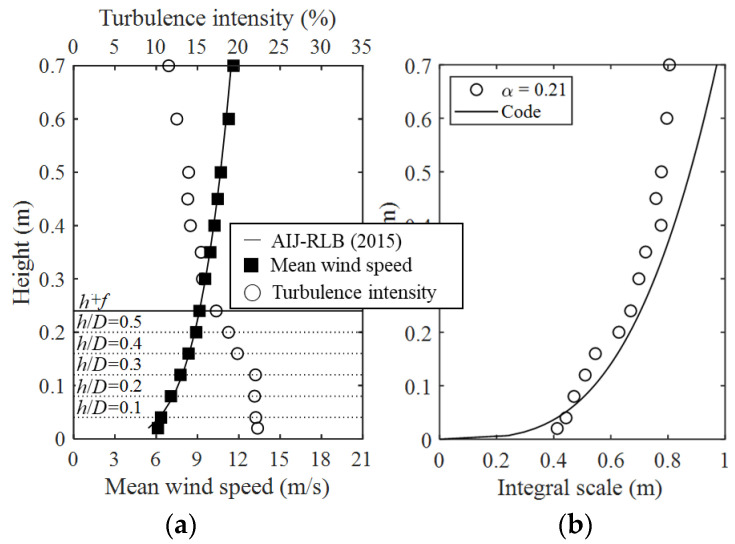
Profiles of experimental flows: (**a**) mean wind speed and turbulence intensity and (**b**) turbulence length scale.

To reproduce the oncoming flows, spires, barriers, and roughness blocks were used to reproduce the power-law index α = 0.21 (semi-urban area) conditions in the wind tunnel. Figure 5 shows the graphs of the mean wind speed, turbulence intensity, and turbulence length scale of the oncoming flows. At the maximum height (*H + f*), which corresponds to *h*/*D* = 0.5 in Figure 5a, the mean wind speed was 9.1 m/s and the turbulence intensity was 17.3%. The turbulence length scale in Figure 5b was calculated using Equation (1).

(1) $$L_{ux}=\int_{0}^{\infty }\tilde{R_{12}}\left(r\right)dr$$

**Figure 6:**
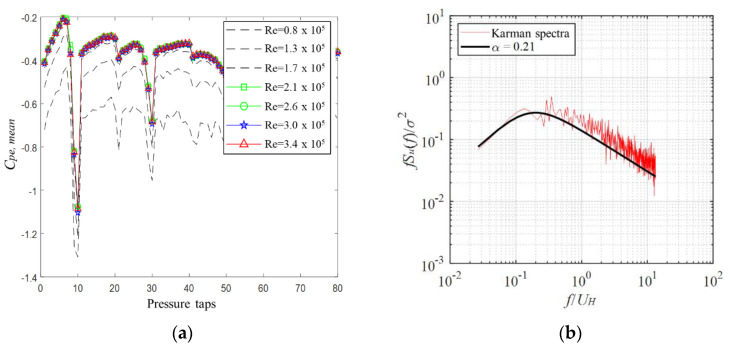
Reynolds number and power spectra: (**a**) mean pressure coefficients at different Reynolds numbers. (**b**) Power spectra of velocity fluctuations.

The flow was set to the wind speed and was not affected by the Reynolds number [15,16]. Figure 6a shows the mean peak pressure coefficients as a function of the number of pressure taps at different Reynolds numbers. The *x*-axis indicates the total number of pressure taps, and the *y*-axis indicates the wind pressure coefficient. The change in the wind pressure coefficient is stable without any significant differences from its value at a Reynolds number of Re = 2.1 × 10^5^ (green box in the legend). Therefore, we set Re = 2.4 × 10^5^, which is similar to the Reynolds number determined by Noguchi and Uematsu [17]. Letchford and Sarkar [18] confirmed that wind pressure distribution is stable within a Reynolds number range of 2.3 × 10^5^–4.6 × 10^5^. Figure 6b shows the power spectra of wind velocity fluctuations for the two oncoming flows at the maximum model height of 0.24 m. These spectra are consistent with the Karman spectra. 

Since this paper needed a basis of comparison, the mean wind speed and turbulence intensity were measured through multiple simulations using Spires, barriers, and roughness blocks to match the airflow conditions in the wind tunnel in detail with the AIJ–RLB (2015) standard. Furthermore, in the case of a spherical dome roof, the wind pressure distribution varies according to the Reynolds number, so many related prior studies were referred to and the Reynolds number with the most stable wind pressure change was implemented. Therefore, it seems reasonable to compare the experimental results with the standard. 

## 3. Results and Discussion

The time history of wind pressure is calculated by C*_p_(t) =* (*P*-*P_pitot_*)/*q_H_*, where *P* is the pressure measured at each pressure tap; *P_pitot_* is the pressure measured in the pitot tube installed 1.2 m above the wind tunnel floor, and *q_H_* is the velocity pressure at the maximum roof height (*H* + *f)* for each model (see Figure 2). The peak pressure coefficients proposed in the Japanese wind load code (AIJ–RLB (2015)) were defined as the negative and maximum values for each 10-min sample of C*_p_(t)*. Moreover, 10 ensemble-averaged values were calculated for the external peak pressure coefficients to be applied in the cladding design. Therefore, for a more accurate comparison, this study analyzed 10 ensemble-averaged values under the same conditions.

**Figure 7:**
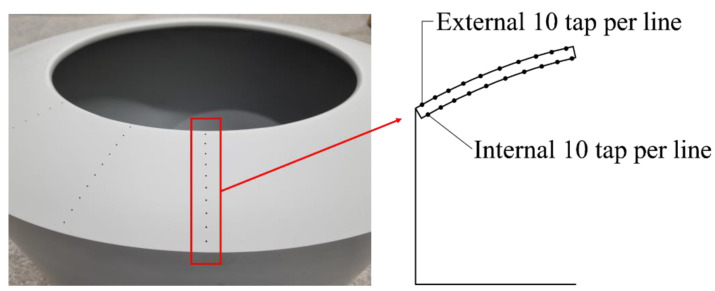
Points of pressure collected.

The data obtained from the wind tunnel test are utilized as the peak pressure coefficients for the cladding design. These coefficients include a negative external peak pressure (*C_pe,min_*), a positive external peak pressure (*C_pe,max_*), an inner surface negative internal peak pressure (*C_pi,min_*), and an inner surface positive internal peak pressure coefficients (*C_pi,max_*) (see Figure 7). The estimations of the wind pressure coefficients are achieved using Equations (2)–(5).

(2) $$C_{Pe,min}=\frac{P_{e,min}}{q_{Href}}$$

(3) $$C_{Pe,max}=\frac{P_{e,max}}{q_{Href}}$$

(4) $$C_{Pi,min}=\frac{P_{i,min}}{q_{Href}}$$

(5) $$C_{Pi,max}=\frac{P_{i,max}}{q_{Href}}$$

$P_{e,min}$ and $P_{e,max}$ are the minimum and maximum wind pressure values, respectively, for each pressure tap on the external roof surface. Similarly, $P_{i,min}$ and $P_{i,max}$ are the minimum and maximum values of the internal roof surface, respectively. In addition, $q_{Href}$ is the design velocity pressure at the maximum height (*H* + *f*) for each dome roof height.

The peak net pressure coefficients were calculated using Equation (6) at the same locations of the lines and pressure taps installed on the external roof surface and internal roof surface.

(6) $$C_{pn,i}=C_{pe,i}-C_{pi,i\;},$$

where the difference between the external roof surface pressure $C_{pe,i}$ and internal roof surface pressure $C_{pi,i}$ is the peak net pressure coefficient $C_{pn,i}$. This study defines the minimum and maximum values for *C_pn,i_* as the negative peak net pressure coefficient (*C_pn,min_*) and positive peak net pressure coefficient (*C_pn,max_*), respectively.

Generally, when calculating the external peak pressure coefficient, the extreme value, estimated using the Cook–Mayne method [19], is used to enhance the reliability of the statistics. By using the peak values of the 10 samples, the extremes are calculated according to the mode and dispersion of the Fisher–Tippett (Type 1) distribution by formulating the best linear unbiased estimator (BLUE) [20]. Additionally, to compare these values with both the results of previous studies and the wind load codes of different countries, a non-exceedance probability of the Gumbel distribution of 78% is typically used.

However, this study defined the external peak pressure coefficients as the minimum and maximum values for each averaged sample (over a 10-min period) and calculated the 10 ensemble-averaged values for the external peak pressure coefficient applied to the cladding design. To verify the difference between the 10 ensemble-averaged values and extreme values, the values were derived based on the two methods and compared. 

**Figure 8:**
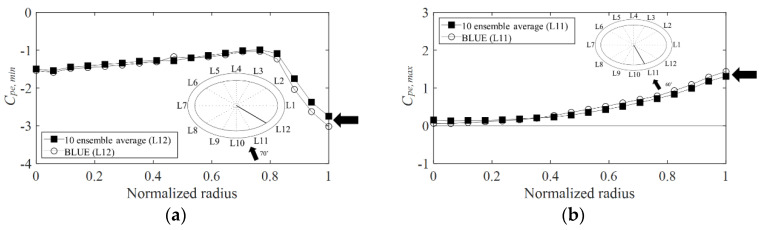
Comparison between 10 ensemble-averaged and BLUE values at the largest value derived taps: (**a**) *C_pe,min_* and (**b**) *C_pe,max_*.

Lee et al. [1] analyzed the wind pressure coefficients of an elliptical retractable dome roof and compared the ensemble average and extreme values derived with BLUE. Figure 8 shows the results. The graphs compare the ensemble averages and extreme values derived with BLUE. This is an example of the negative and positive external peak pressure coefficients in the line of the pressure taps exhibiting the largest absolute value in all the wind directions of the roof. The *x*-axis shows the normalized radius, and the *y*-axis shows the wind pressure coefficient. According to a comparison of the absolute value difference between the ensemble-averaged values (boxes) and BLUE extreme values (circles), the difference is approximately 10%. However, the tendencies of variation in the absolute value are extremely similar. Additionally, the wind pressure coefficients proposed in the Japanese wind load code (AIJ–RLB (2015)) are data obtained from the 10 ensemble-averaged values. Therefore, for a more accurate comparison, the 10 ensemble-averaged values are selected and analyzed in this study.

### 3.1. Peak Pressure Coefficient Characteristics of Elliptical Center-Open Dome

**Figure 9:**
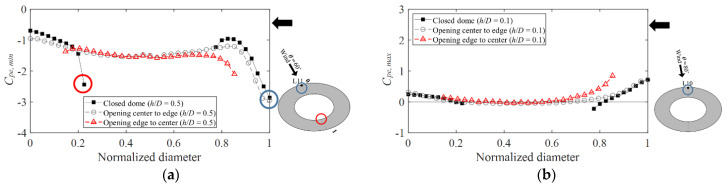
Comparison of opening roof and closed roof: (**a**) *C_pe,min_* (*h/D =* 0.5) and (**b**) *C_pe,max_* (*h/D =* 0.1).

Unlike that of dome roofs that open from the edge to the center, the opening of dome roofs that open from the center to the edge is located in the center. Figure 9a compares a closed roof with both a dome roof that opens from the center to the edge and that which opens from the edge to the center. For *C_pe,min_*, the comparison was made at *h*/*D* = 0.5, where the absolute values were the largest (*h*/*D* = 0.1 for *C_pe,max_*). The analysis expresses the line of the pressure taps from which the largest absolute value was derived in all the wind directions. The *x*-axis shows the normalized diameter, and the *y*-axis shows each peak pressure. A normalized diameter of 1 represents the windward side, 0.5 represents the center of the dome, and 0 represents the leeward side.

On the windward side around a normalized diameter from 0.8 to 1, the trends of the absolute value and variation were similar to those of the closed roof owing to the effects of separation and reattachment. In contrast, because of the open space in the roof that opens from the edge to the center, there is no direct vortex influence owing to separation. Thus, the flow becomes complex, and the absolute value decreases significantly compared with those of a closed roof and a roof that opens from the center to the edge owing to turbulence. On the leeward side, air flows along the roof at the central area of the dome. Within a circular region at an approximate normalized diameter of 0.2, the absolute value increases abruptly, similar to the windward side. During this phenomenon, the flow that is separated from the windward roof surface is separated again at the edge of the dome area. 

Considering the positive external peak pressure coefficients in Figure 9b, the locations of the separation varied because the end-of-roof locations were different. However, no significant change occurs in terms of their absolute values.

**Figure 10:**
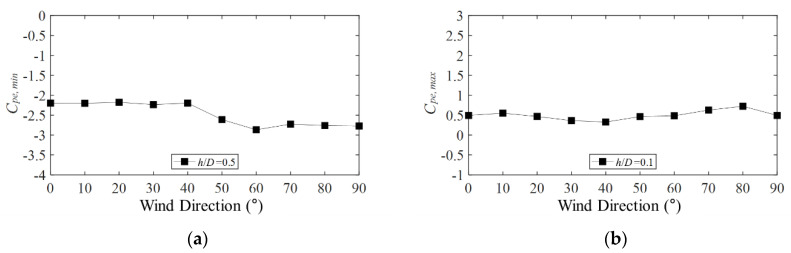
Plots of largest absolute values as a function of wind direction: (**a**) *C_pe,min_* and (**b**) *C_pe,min_*.

Figure 10a shows the negative external peak pressure coefficients for each wind direction at *h*/*D* = 0.5. The peak net pressure coefficients are calculated for the same line and pressure taps of the outer and inner surfaces of the roof in the same time history. Accordingly, to determine the wind direction from which the largest absolute value is derived to analyze the peak net pressure coefficient, the absolute value of the largest wind pressure coefficient among the pressure taps in the various wind directions was derived. According to the analyzed results, the absolute value did not change until the wind direction was 40°. However, it increased when the wind direction was 50°, which could be attributed to the longitudinal and transverse axes and the change in the representative length with the axis direction, thus causing the boundary layer formation to change and the wind pressure coefficients to vary. For the positive external peak pressure coefficients in Figure 10b, the absolute values for each wind direction did not differ considerably.

### 3.2. External Roof Surface Peak Pressure Coefficients

**Figure 11:**
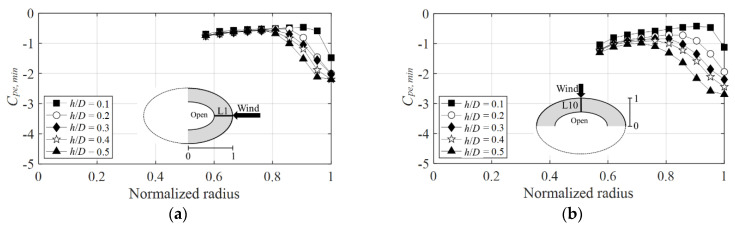
Variation of *C_pe,min_* according to wind direction (windward side): (**a**) 0° and (**b**) 90°.

Figure 11 shows the negative external peak pressure coefficients for the wind directions of 0° and 90° on the windward side. The *x*-axis is the normalized radius, and the *y*-axis is the negative external peak pressure coefficient. Herein, a normalized radius of the *x*-axis equal to 0 represents the center of the dome, and a value equal to 1 represents the windward side. 

**Figure 12:**
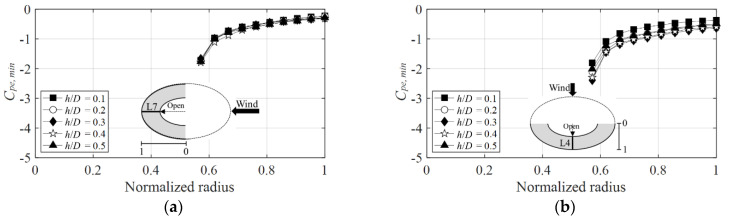
Variation of *C_pe,min_* according to wind direction (leeward side): (**a**) 0° and (**b**) 90°.

Figure 11a shows that the largest negative external peak pressure coefficient is attained near the normalized radius equal to 1 where separation occurs, and its value when the wind direction is 0° is −2.2 at *h*/*D* = 0.5. Figure 11b shows that, at the same *h*/*D*, the negative external peak pressure coefficient is attained when the value at the wind direction of 90° is −2.7, with an absolute value greater than that at the wind direction of 0°. Figure 12 shows the negative external peak pressure coefficients at the wind directions of 0° and 90° on the leeward side. The negative external peak pressure coefficient on the leeward side shows a larger absolute value at a wind direction of 90° (which equals −2.4, see Figure 12a) than that at a wind direction of 0° (which equals −1.7, see Figure 12b). Notably, no variation occurred again in the absolute value according to changes in *h*/*D* at the point where the center of the open dome was separated, which was near a normalized radius of 0.6. Following reattachment on the windward side, the flow characteristics were similar owing to the influence of the boundary layer formed on the roof surface, thus resulting in similar values regardless of the changes in *h*/*D* on the leeward side. 

**Figure 13:**
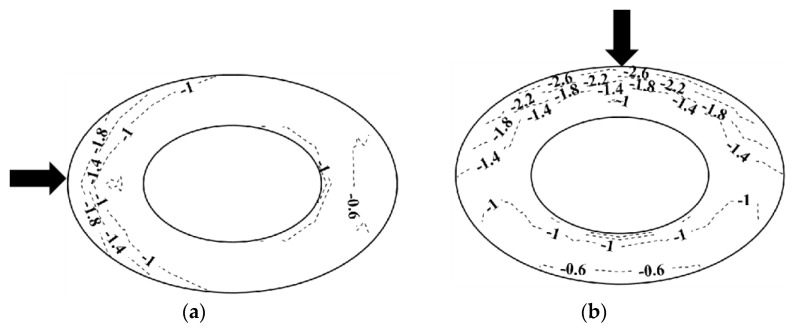
Distribution of *C_pe,min_*: (**a**) 0° and *h*/*D* = 0.5 and (**b**) 90° and *h*/*D* = 0.5.

Figure 13 shows the distribution of the negative external peak pressure coefficient at *h*/*D* = 0.5 of the dome roof showing the largest absolute value in the two wind directions. The distribution of the largest absolute value of the negative external peak pressure coefficient was typical on the windward side, regardless of the height, and was larger at 90°, attributed to turbulence deformation. For smaller objects, the vortex passes through with almost no change in shape, whereas for larger objects, it is split into large vortices. Hence, the influence of the larger vortex was in the 90° wind direction. Moreover, the negative external peak pressure coefficient increased with an increase in diameter. Moreover, the negative external peak pressure coefficient increased with an increase in diameter.

When there is a longer axis such as an elliptical dome roof, the Reynolds number changes in proportion to the length in the axial direction, so the boundary-layer formation changes depending on the wind direction and the negative peak pressure coefficients are greater.

**Figure 14:**
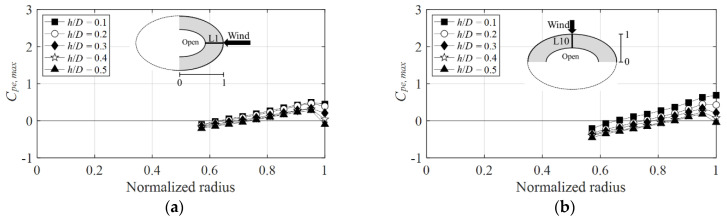
Variation of *C_pe,max_* according to wind direction (windward side): (**a**) 0° and (**b**) 90°.

**Figure 15:**
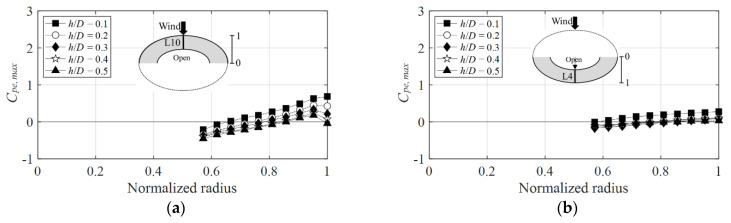
Variation of *C_pe,max_* according to wind direction (leeward side): (**a**) 0° and (**b**) 90°.

Figure 14 shows the positive external peak pressure coefficients as a function of the normalized radius for the wind directions of 0° and 90° on the windward side. The positive external peak pressure coefficient shows the greatest absolute value at *h*/*D* = 0.1, and the absolute value tends to decrease as *h*/*D* increases. Figure 14a shows that the largest positive external peak pressure coefficient near the normalized radius is equal to 1, and at a wind direction of 0° is 0.5 at *h*/*D* = 0.1, and the coefficient at the 90° wind direction is 0.7 (Figure 14b). Thus, the positive external peak pressure coefficient was larger at 90° than that at 0°, although the difference was not significant. A similar trend was observed on the leeward side in Figure 15. The reason that the difference in absolute values of positive peak pressure coefficients is not large depending on the wind direction is that static pressure is affected by turbulence, and there is no significant effect because the rise of the roof is low.

### 3.3. Peak Pressure Coefficients and Peak Net Pressure Coefficients of Internal Roof Surface

According to the experimental results, given that positive pressure did not occur inside the dome roof, only the negative external peak pressure coefficients were analyzed for the inner surface [21]. This study also analyzed the peak net pressure coefficient, which is the pressure difference between the outer and inner surfaces.

**Figure 16:**
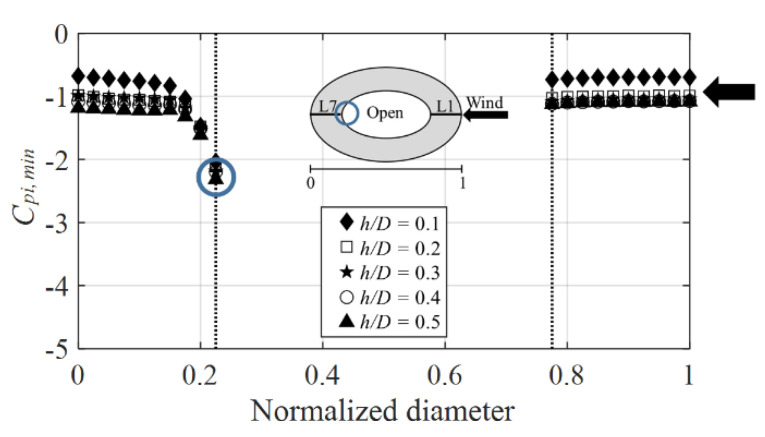
Variation of *C_pi,min_*.

Figure 16 shows the graph of the inner surface negative external peak pressure coefficients at the line showing the largest absolute value in the various wind directions. The *x*-axis displays the normalized diameter and the *y*-axis the wind pressure coefficient. A normalized diameter 1 is on the windward side, 0.5 at the center of the dome, and 0 on the leeward side. At all the *h*/*D* values, the absolute value increased considerably and was distributed from 2.0 to 2.2 owing to the separation that occurred at the roof end at the center of the dome near a normalized diameter of 0.2 (blue circle), similar to that at the roof end on the outer surface. In contrast, on the windward side, the absolute value was constant. However, at *h*/*D* = 0.1, the absolute value was relatively smaller than that at *h*/*D* = 0.2–0.5 because the roof height interacted with the ground surface.

**Figure 17:**
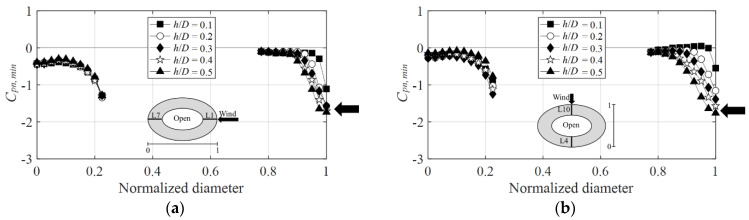
Variation of *C_pn,min_* according to wind direction: (**a**) 0° and (**b**) 90°.

Figure 17 shows the graphs of the negative peak net pressure coefficients at the wind directions of 0° and 90°. Figure 17a shows the negative peak net pressure coefficient at the 0° wind direction. The peak net pressure coefficients of both the windward and leeward sides were smaller than the individual negative external peak pressure coefficients at the outer and inner sides of the roof. Herein, the negative pressures generated on the roof’s outer side and inner side offset each other, thus decreasing the absolute value of the negative peak net pressure coefficient. 

Figure 17b shows the negative peak net pressure coefficients at the 90° wind direction. On the windward side, these values and the changes are similar to those in the 0° wind direction. However, on the leeward side, they are smaller because the representative length changes with the wind direction. As the wind direction increases to 90°, the roof length shortens, thus decreasing the thickness of the boundary layer at the center of the dome and increasing the negative pressure. Thus, considering that the negative pressure on the outer surface increases by a greater extent than that along the 0° wind direction, the negative pressures on the outer and inner sides further offset each other, thereby decreasing the negative peak net pressure coefficient of the leeward side.

**Figure 18:**
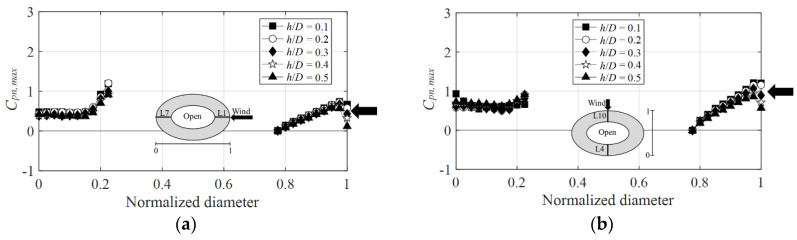
Variation of *C_pn,max_* according to wind direction: (**a**) 0° and (**b**) 90°.

Figure 18 shows the graphs of the positive peak net pressure coefficients at the wind directions of 0° and 90°. The absolute value at the wind direction of 0° on the leeward side (Figure 18a) was somewhat larger than that of the 90° wind direction (Figure 18b) because, as mentioned previously, the representative length decreases owing to the change in the wind direction, thus causing the negative pressure in the center of the dome to increase. Additionally, the positive peak net pressure coefficient on the windward side was smaller than the positive external peak pressure coefficient on the outer side of the roof. Moreover, on the leeward side, the positive external peak pressure coefficient on the outer side of the roof was greater. The same reason is applied to the negative peak net pressure coefficient. this is attributed to the dominant negative pressure and minimal influence of the positive pressure. On the inner side of the roof, the influence of the negative pressure increases owing to separation, thus leading to an upward pressure due to the wind pressure difference.

### 3.4. Comparison of Experimental Values for Different Wind Load Codes

This study used the internal peak pressure coefficients of the Korean wind load code (KDS 41 10 15) to compare the peak net pressure coefficients of the center-open dome roof. For internal peak pressure coefficients, the Japanese wind load code (AIJ–RLB (2015)) proposes distinct values for positive and negative pressures, whereas the Korean wind load code (KDS 41 10 15) proposes values for two cases (with and without a dominant opening) in details. Table 2 shows the internal peak pressure coefficients proposed by the Japanese (AIJ–RLB (2015)) and Table 3 shows Korean wind load codes (KDS 41 10 15).

#### Peak Net Pressure Coefficients

**Figure 19:**
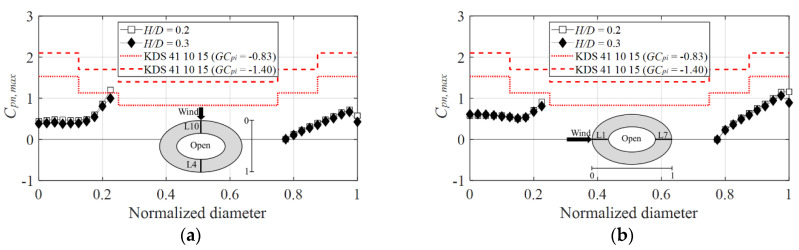
Comparison of positive peak net pressure coefficients according to AIJ–RLB (2015): (**a**) 0° and (**b**) 90°.

Figure 19a graphically compares the positive peak net pressure coefficients of both codes at the 0° wind direction. The influence of the negative pressure due to the separation on the inner surface and leeward side at normalized diameter values in the proximity of 0.2 was greater than that on the outer side. Thus, the negative pressure was not offset, and the negative peak net pressure coefficient exceeded the positive peak net pressure coefficient calculated based on the value of 0.83 (dotted line) by at most 1.1 times. In contrast, at the 90° wind direction, as shown in Figure 19b, the values indicated by both codes were consistent. 

**Figure 20:**
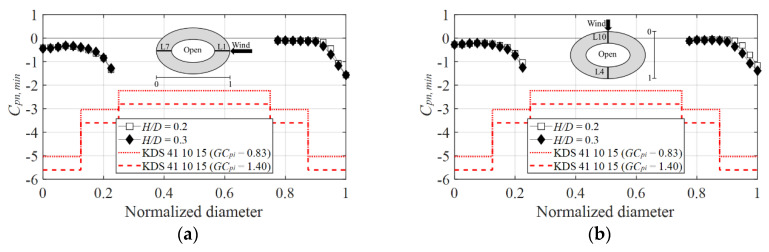
Comparison of negative peak net pressure coefficients with AIJ–RLB (2015): (**a**) 0° and (**b**) 90°.

Considering the negative peak net pressure coefficients, as the negative pressure is dominant on the inner surface at both the 0° (Figure 20a) and 90° (Figure 20b) wind directions, it offsets the negative pressure generated on the outer surface, confirming that both code values were not only satisfied but overestimated.

### 3.5. Comparison with Proposed Peak Pressure Coefficients for Spherical Dome with Opening

Table 4 shows the proposed values by Cheon et al. [7] of the peak net pressure coefficients for cladding designs obtained via wind tunnel tests of a center-open retractable circular dome roof. The tests were conducted with dome roof opening ratios of 30% and 50% (opened length defined as the opening ratio), *f*/*D* = 0.1, *h*/*D* = 0.1, 0.2, 0.3, 0.4, and 0.5, power-law index α = 0.21, and a moving average time of 1 s. The same experimental conditions were used in this study.

This study compared the proposed values of the 50-% circular dome roof for the models with opening ratios of 30% and 50% because it is more similar to the roof area with an opening ratio of 30% in the model tested in this study. 

As shown in Table 4, the area proposed in the previous study was divided into two zones: For Zone 1, five R_a_ values were proposed for all the *h*/*D* in the area corresponding to 60% of the dome roof end. For Zone 2, one value of R_b_ was proposed for all the *h*/*D* values in the area corresponding to the remaining 40% of the dome roof.

**Figure 21:**
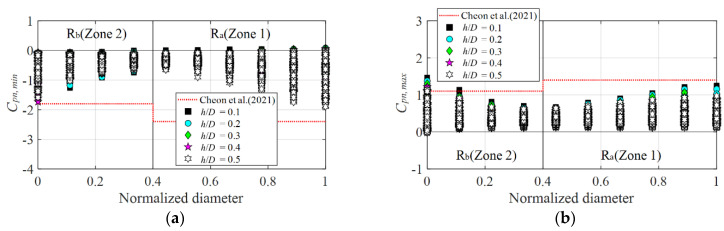
Comparison of peak net pressure coefficients with previous research: (**a**) *C_pn,min_* and (**b**) *C_pn,max_*.

Figure 21 shows the comparison of the proposed values for the circular dome roof in the previous study (with a 50-% opening ratio), *h*/*D* = 0.4 (the height at which the largest negative peak net pressure coefficient was obtained) and *h*/*D* = 0.1 (the height where the largest positive peak net pressure coefficient was obtained), with the negative and positive peak net pressure coefficients at the experimental values in this study, *h*/*D* = 0.1, 0.2, 0.3, 0.4, and 0.5. Figure 20a shows that the negative peak net pressure coefficients yielded similar trends with no significant differences between the proposed values and experimental values in both the R_a_ and R_b_ zones at all the *h*/*D* values. In contrast, for the positive peak net pressure coefficients, as shown in Figure 20b, the experimental values at all the *h*/*D* exceeded the proposed values by at most 1.3 times in the central zone of the dome, R_b_.

### 3.6. Proposal of Peak Net Pressure Coefficients

According to the above-mentioned analysis, for a center-open dome roof with a 30-% opening ratio, it was appropriate to use the proposed values of the previous study for the negative peak net pressure coefficients as they satisfied the code at all conditions according to a comparison with the proposed values for a circular dome roof of Cheon et al. [7]. 

For the positive peak net pressure coefficients, the experimental values at all the *h*/*D* exceeded the proposed values in only the central zone of the dome, R_b_, and satisfy the code in the end zone of the dome, R_a_. Therefore, a code was proposed based on the experimental values only in the central zone of the dome, R_b_, and was limited to the positive peak net pressure coefficient. Additionally, in the previous study, as similar R_b_ values were observed regardless of changes in *h*/*D* at the central zone of the dome, only one positive peak net pressure coefficient was proposed, whereas the absolute values of the experimental values in this study tended to change according to changes in *h*/*D* because the representative length (length of the open space) varying with changes in the wind direction, with the negative external peak pressure coefficient on the inner surface changing accordingly.

Therefore, negative peak net pressure coefficients for each *h*/*D* were proposed as shown in Table 5. Similar to that of the previous study, the proposed area was divided into two zones. 

**Figure 22:**
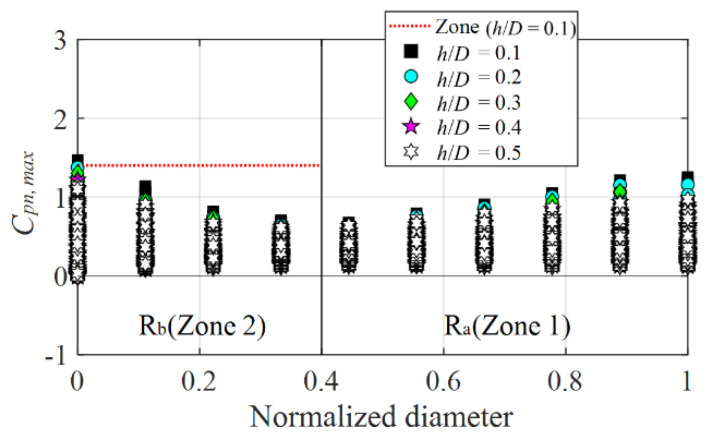
Proposed positive peak net pressure coefficients for elliptical dome with an opening ratio of 30%.

Figure 22 shows the proposed positive peak net pressure coefficients for each *h*/*D* in the zones of a center-open retractable elliptical dome roof with an opening ratio of 30%. The figure shows the absolute value of the pressure tap (*C_pn,max_*), indicating the largest absolute value in the various wind directions. The red dotted line corresponds to *h*/*D* = 0.1, indicating the largest absolute value among all the *h*/*D* values. The proposed R_b_ values range from 1.2 to 1.4 for each *h*/*D* in the central zone of the dome.

## 4. Conclusions

This study proposed a code for the cladding design of elliptical dome roofs based on an analysis of wind pressure characteristics and a comparison of existing codes of center-open elliptical dome roofs. The external peak pressure and peak net pressure coefficients analyzed based on wind tunnel tests were compared with the proposed external peak pressure coefficients for retractable circular dome roofs in a previous study based on the Korean wind load code KDS 41 10 15. The main results are summarized as follows:

(1) The trend of the external peak pressure coefficient on the inner surface of the center-open elliptical dome roof was dominated by the negative pressure, rendering the absolute values of the negative external peak pressure coefficients constant on the windward side. On the leeward side, the values and trends were similar to those of the negative external peak pressure coefficients on the outer surface of the roof owing to separation.
(2) Considering the trends of the peak net pressure coefficient, the negative pressure was offset on the windward side compared with the outer side owing to the influence of the negative pressure on the inner surface, and the absolute value of the peak net pressure coefficient decreased. The positive peak net pressure coefficients were similar to the positive external peak pressure coefficients on the outer and windward sides of the roof. On the leeward side, because the negative pressure significantly increased owing to the separation that occurred on the inner side of the roof, upward pressure was generated owing to the difference in wind pressure. As a result, the positive peak net pressure coefficient was larger than the positive external peak pressure coefficient on the outer side of the roof, and the absolute value of the negative peak net pressure coefficient decreased as the large negative pressures offset each other.
(3) The peak net pressure coefficients were compared with the Korean wind load code KDS 41 10 15. When h/D = 0.3, the experimental values of the positive peak net pressure coefficient exceeded the code by 1.1 times in the R_b_ zone.
(4) The peak net pressure coefficients for the cladding design of center-open elliptical dome roofs were proposed based on the experimental values. For the negative peak net pressure coefficient, a proposal was unnecessary because the values were similar to the proposed values in a previous study for center-open circular dome roofs. The proposed values for the positive peak net pressure coefficient ranged from 1.2 to 1.4 according to the *h*/*D* value in the R_b_ zone. Thus, the same proposed values in the previous study for the R_a_ region could be used.
(5) While the experimental values exceeded the proposed values of the previous study, they exceeded the values in specific areas rather than those in the entire area in some cases. Conversely, they occasionally satisfied the proposed values. For dome roof structures with specific shapes, such as elliptical roofs, appropriate external peak pressure coefficients should be proposed for each area to consider stability and prevent overdesigning.

The result of this study proposes a peak pressure coefficient for cladding design of a retractable dome roof, which currently has no standard worldwide, and is expected to contribute to wind load estimation.

## Figures and Tables

**Table 1:** Summary of numbers of pressure taps.

Pressure Taps			
Line	External Tap	Internal Tap	Total
1	10	10	20
2	10	10	20
3	10	10	20
4	10	10	20
			80

**Table 2:** Internal peak pressure coefficients for cladding design prescribed in AIJ–RLB (2015) [6].

AIJ–RLB (2015) [6]		
partially open buildings	without dominant openings	0 or −0.5

**Table 3:** Internal peak pressure coefficients for cladding design prescribed in KDS 41 10 15 [12].

KDS 41 10 15 [12]		
Closed		0.00 or −0.52
partially open buildings	without dominant openings	+0.83 or −0.83
	dominant openings	+1.40 or −1.40
open		0

**Table 4:** Proposed peak net pressure coefficient for cladding design for dome with an opening ratio of 50% (Cheon et al. [7]).

Negative Peak Net Pressure Coefficients				
*f*/*D*	α	*h*/*D*	Zone 1 (d × 0.6)	Zone 2 (d × 0.4)
0.1	0.21	0.1	−2.0	−1.8
		0.2	−2.3	
		0.3	−2.3	
		0.4	−2.4	
		0.5	−2.1	
Positive peak net pressure coefficients				
*f*/*D*	α	*h*/*D*	zone 1 (d × 0.6)	zone 2 (d × 0.4)
0.1	0.21	0.1	1.4	1.1
		0.2	1.2	
		0.3	1.0	
		0.4	1.0	
		0.5	1.0	
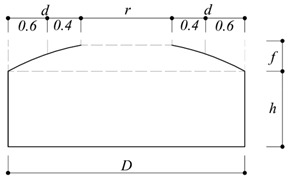				*r*: Opened area *d*: Roof diameter *h*: Wall height *D*: Building diameter *f*: Roof rise

**Table 5:** Proposed peak net pressure coefficient for cladding design for elliptical dome with opening ratio of 30%.

Positive Peak Net Pressure Coefficients			
*f*/*D*	α	*h*/*D*	Zone 1 (d × 0.6)
			*r*/*D* = 0.3
0.1	0.21	0.1	1.4
		0.2	1.4
		0.3	1.3
		0.4	1.2
		0.5	1.2
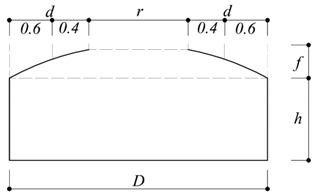			*d*: Roof diameter *h*: Wall height *D*: Building diameter *f*: Roof rise

## Data Availability

Not applicable.

## References

[1] Lee J.H. (2022). Proposal of Peak Pressure Coefficient for Cladding Design of Elliptical Retractable Dome Roof by Wind Tunnel Test. Ph.D. Thesis.

[2] Majowiecki M. (2005). Structural Architecture of Wide Span Enclosures: Uncertainties in Reliability Assessment.

[3] Kumar K.S., Stathopoulos T. (2000). Wind Loads on Low Building Roofs: A Stochastic Perspective. J. Struct. Eng..

[4] Kim Y.S., Hwang G.S., Hwang J.S. Comparison of Wind Pressure Patterns According to Roof Shapes of Stadium. Proceedings of the WEIIK Symposium.

[5] Cheon D.J., Kim Y.C., Yoon S.W. (2018). Comparison of Wind Pressure Coefficient and Wind Load Standard for Cladding in a Retractable Dome roof by Wind Tunnel Test. J. Korean Assoc. Spat. Struct..

[6] Architectural Institute of Japan (AIJ) (2015). AIJ Recommendations for Loads on Buildings.

[7] Kim Y.C., Yoon S.W., Cheon D.J., Song J.Y. (2019). Characteristics of Wind Pressures on Retractable Dome Roofs and External Peak Pressure Coefficients for Cladding Design. J. Wind Eng. Ind. Aerodyn..

[8] Cheon D.J., Kim Y.C., Lee J.H., Yoon S.W. (2021). Experimental Investigation of Wind Pressure Characteristics for Cladding of Dome Roofs. Materials.

[9] Park M.J., Yoon S.W., Kim Y.C., Cheon D.C. (2022). Wind Pressure Characteristics Based on the Rise-Span Ratio of Spherical Domes with Opening on the Roof. Buildings.

[10] Lee J.H., Kim Y.C., Cheon D.J., Yoon S.W. (2020). Analysis of External Peak Pressure Coefficients for Cladding in Elliptical Retractable Dome Roof by Wind Tunnel Test. J. Korean Assoc. Spat. Struct..

[11] Lee J.H., Kim Y.C., Cheon D.J., Yoon S.W. (2021). Wind Pressure Characteristics of Elliptical Retractable Dome Roofs. J. Asian Architect. Build. Eng..

[12] (2019). Building Structure Standards.

[13] Ishii K. (2000). Structural Design of Retractable Roof Structures.

[14] Yoshida A., Tamura Y., Kurita T. (2001). Effects of Bends in a Tubing System for Pressure Measurement. J. Wind Eng. Ind. Aerodyn..

[15] Sun Y., Qiu Y., Wu Y. (2013). Modeling of Wind Pressure Spectra on Spherical Domes. Int. J. Space Struct..

[16] Cheng C.M., Fu C.L. (2010). Characteristic of Wind Loads on a Hemispherical Dome in Smooth Flow and Turbulent Boundary Layer Flow. J. Wind Eng. Ind. Aerodyn..

[17] Noguchi M., Uematsu Y. Model of Fluctuating Wind Pressures on Spherical Domes for Load Estimation of Cladding. Proceedings of the 18th National Symposium on Wind Engineering.

[18] Letchford C.W., Sarkar P.P. (2000). Mean and Fluctuating Wind Loads on Rough and Smooth Parabolic Domes. J. Wind Eng. Ind. Aerodyn..

[19] Cook N.J., Mayne J.R. (1980). A Refined Working Approach to the Assessment of Wind Loads for Equivalent Static Design. J. Wind Eng. Ind. Aerodyn..

[20] (1974). Efficient Methods of Extreme-Value Methodology.

[21] Xu H., Lou W. (2018). Wind-Induced Internal Pressures in Building with Dominant Opening on Hemi-Ellipsoidal Roof. J. Eng. Mech..

